# Feature Selection and Dwarf Mongoose Optimization Enabled Deep Learning for Heart Disease Detection

**DOI:** 10.1155/2022/2819378

**Published:** 2022-12-07

**Authors:** S. Balasubramaniam, K. Satheesh Kumar, V. Kavitha, A. Prasanth, T. A. Sivakumar

**Affiliations:** ^1^Department of Futures Studies, University of Kerala, Thiruvananthapuram, Kerala, India; ^2^Department of Computer Science and Engineering, University College of Engineering, Kanchipuram, Tamil Nadu, India; ^3^Department of ECE, Sri Venkateswara College of Engineering, Sriperumbudur, Tamilnadu, India; ^4^Faculty of Engineering and Technology, Villa College, Male, Maldives

## Abstract

Heart disease causes major death across the entire globe. Hence, heart disease prediction is a vital part of medical data analysis. Recently, various data mining and machine learning practices have been utilized to detect heart disease. However, these techniques are inadequate for effectual heart disease prediction due to the deficient test data. In order to progress the efficacy of detection performance, this research introduces the hybrid feature selection method for selecting the best features. Moreover, the missed value from the input data is filled with the quantile normalization and missing data imputation method. In addition, the best features relevant to disease detection are selected through the proposed hybrid Congruence coefficient Kumar–Hassebrook similarity. In addition, heart disease is predicted using SqueezeNet, which is tuned by the dwarf mongoose optimization algorithm (DMOA) that adapts the feeding aspects of dwarf mongoose. Moreover, the experimental result reveals that the DMOA-SqueezeNet method attained a maximum accuracy of 0.925, sensitivity of 0.926, and specificity of 0.918.

## 1. Introduction 

Heart disease destroys the function and structure of the heart, which causes the major death of humans around the globe. Several heart diseases produce heart attacks, the most difficult cardiovascular disease [1]. The major part of the human body is the heart, which pumps blood into the entire body organ. In case, the heart does not function properly, then the different organs in the human body will stop to work make to death. Hence, the regular functioning of the heart is very important. Heart disease is considered the most important reason for death worldwide [2]. Moreover, heart disease is generally occurring in both women and men. Hence, the invention of an efficient heart disease prediction technique helps to reduce the death rate [3, 4]. In the medical field, heart disease diagnosis is a complex task, regularly increasing the mortality rate. Hence, the researchers have introduced an automatic disease diagnosis technique for perceiving heart disease. To detect heart disease, the researchers gathered the clinical data from clinical experience, and the detection is done by decision-making method and doctor's diagnosis [1].

Recently, various researchers utilized machine learning, data mining, and deep learning techniques in healthcare for predicting heart disease [5]. Deep learning is an extended version of the machine learning model, normally utilized in image and data processing techniques in numerous medical fields [6–9]. Normally, data mining methods are utilized to compute the relationship among numerous factors and hidden information of input data [10, 11]. Various deep learning models [12] have been applied to acquire the significant performance of heart disease prediction [13, 14]. For heart disease prediction, feature selection is considered as a significant step [13, 15]. The high reliability and precision classification methods offer more assistance to data in recognizing prospective patients. The commonly used heart disease prediction techniques are logistic regression, clustering algorithm, Naïve Bayes, neural networks, and support vector machine (SVM), which offer substantial performance in heart disease prediction [16, 17]. Furthermore, missing and uncertain data disturbs the prediction method's performance [4, 18]. Moreover, deep learning methods provide effective performance with massive and unclear datasets. In addition, deep learning techniques help to classify the inexistence and existence of heart disease [2, 19].

The heart disease prediction technique using a newly devised model is explained in this paper. The input data is preprocessed here using quantile normalization and missing data imputation. Then, the preprocessed data is processed under the feature selection to choose the relevant features based on the congruence coefficient and Kumar–Hassebrook similarity. The SqueezeNet does the heart disease prediction, wherein the weight of SqueezeNet is learned by the DMOA that provides the detected outcome is either normal or abnormal patients.

The novelty of this research is specified by

- *Proposed hybrid Congruence coefficient Kumar–Hassebrook similarity for feature selection*: In this research, the best feature from the input data is chosen by the hybrid congruence coefficient Kumar–Hassebrook similarity. Here, the preprocessed data is first given to the Congruence coefficient that selects the top score features. Then, these features are again sent to the Kumar-Hassebrook similarity that selects the most appropriate features. In addition, the heart disease prediction is completed by the SqueezeNet, which is learned by the DMOA.

The structure of this paper is given in this section.  Section 2 describes the literature survey of heart disease detection, the proposed methodology is explained in Section 3, results and discussion of the introduced model are exhibited in Section 4 and then Section 5 shows the conclusion of this paper.

## 2. Literature Survey

The survey of numerous heart disease prediction methods is given as follows: Kora et al. [20] introduced the bacterial foraging particle swarm optimization (BF-PSO) for detecting heart disease. Here, the hybrid BF-PSO is designed by integrating Bacterial foraging optimization (BFO) with particle swarm optimization (PSO). Although the proposed model provides improved detection accuracy by extracting more relevant features, this method had maximum training time. Manur et al. [4] modeled the bi-directional long short-term memory with conditional random field (Bi-LSTM-CRF) to predict heart disease. Here, the medical data was examined by the bidirectional LSTM, and the CRF model was employed to compute the relationship among various features. The computation cost of this method was maximum. Budholiya et al. [21] introduced the XGBoost mode for diagnosing heart disease. However, the model failed to process complicated datasets. Oliver et al. [1] introduced the regressive learning-based neural network classifier (RLNNC) for predicting heart disease. This method provided a better detection result, but the computation cost of this method was high.

## 3. Challenges

The complications of various prevailing heart disease prediction techniques are given as follows:

- In [20], the BF-PSO model was introduced to predict heart disease. However, the scheme produced the minimum detection accuracy with very large databases.
- In [4], the bi-LSTM-CRF model was devised to detect heart disease early. However, this method provides poor detection performance since the detection method did not utilize any algorithm for training the classifier.
- The challenges of the proposed method in [21] are that it only detects heart disease, but did not detect any other similar tasks.
- The major challenging step of heart disease prediction is feature extraction. Moreover, using high dimensional data increases the training time of classifiers.

## 4. Proposed Congruence Coefficient Kumar-Hassebrook Enabled Feature Selection and DMOA-SqueezeNet for Heart Disease Detection

**Figure 1:**
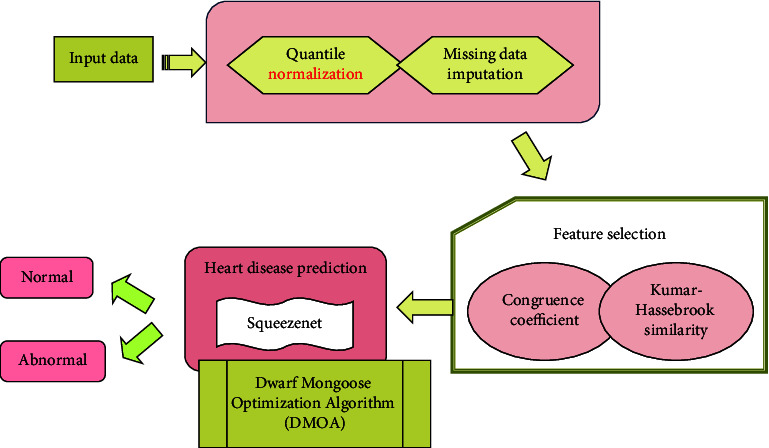
Block diagram of the proposed technique for heart disease detection.

This research introduced an effective heart disease detection approach, namely, DMOA-SqueezeNet. Initially, input data is considered from a specific dataset [22], which is given to preprocessing phase where the image is preprocessed using quantile normalization [23] and missing data imputation. After that, feature selection is done to select the suitable features utilizing the proposed hybrid feature selection scheme, namely, the congruence coefficient Kumar–Hassebrook similarity. Finally, heart disease prediction is performed using SqueezeNet [22], which is trained using an optimization algorithm, namely, DMOA [24]. The block diagram of the newly modeled heart disease detection technique is revealed in Figure 1.

### 4.1. Get the Input Data

The input data is taken from the heart disease dataset *C*, which consists of *d* number of heart disease data, and is formulated as

(1) $$\begin{array}{c} C=\left\{C_{1},C_{2},\ldots ,C_{a},\ldots ,C_{d}\right\}\text{,} \end{array}$$

where *d* denotes the total number of medical data, *C*_*a*_ specifies the *a*^*th*^ number of data, and this data is considered for forecasting the heart disease in this research, and the dimension of original data is *k* × *n*.

### 4.2. Preprocessing

This step explains the preprocessing of input data *C*_*a*_ with size *k* × *n* using quantile normalization [23] and missing data imputation. The preprocessing method is used to remove the redundant data from the input data *C*_*a*_ with size *k* × *n*.

#### 4.2.1. Quantile Normalization

For quantile normalization, the input data (*C*_*a*_)_*k*×*n*_ is subjected to quantile normalization [23] for normalizing it. The process of quantile normalization is a simple procedure to normalize the input data. To perform the quantile normalization, the first step is to rank the input data based on its magnitude values and then compute the average values of input data with the same rank. After that, applying the values of input data occupying that specific rank with the average value. The final step is to rearrange the input data into the original order. Hence, the outcome of quantile normalization is indicated as *Q*_*k*×*n*_.

#### 4.2.2. Missing Data Imputation

After the quantile normalization, the missing data imputation is performed, replacing the missing data from the normalized data *Q*_*k*×*n*_ with the substituted values. Here, the missing values are substituted in two ways, like numerical attribute substitution and categorical attribute substitution. For numerical attribute substitution, the mean values of numerical data are computed and then substituted it with the missing values. For categorical attribute substitution, most data type is substituted with the missing values. Hence, the outcome of missing data imputation is indicated as *X*_*k*×*n*_.

### 4.3. Feature Selection

After the preprocessing, the processed data contains various relevant and irrelevant features. However, all of these features are not necessary for heart disease prediction; hence the prediction process requires only meaningful features. Thus, feature selection is required to select the appropriate and meaningful features. In this research, the suitable features are selected by the proposed hybrid congruence coefficient Kumar–Hassebrook similarity. For that, initially, the preprocessed data *X*_*k*×*n*_ is sent to the congruence coefficient and then the outcome of the congruence coefficient is passed to the Kumar–Hassebrook similarity so that the best features are selected.

#### 4.3.1. Congruence Coefficient

The congruence coefficient [25] is utilized to select the features from *X*_*k*×*n*_ by comparing the candidate feature with the target values. It is used to evaluate the similarity of two configurations. It increases the prediction accuracy of the model. Hence, the expression for the congruence coefficient is given by

(2) $$\begin{array}{c} J_{k}=\frac{\sum \text{PR}}{\sqrt{\sum P^{2}\sum R^{2}}}\text{,} \end{array}$$

where *P* denotes the candidate feature and *R* specifies the target values. After calculating the congruence coefficient, the top *o* features with a high degree of factor are selected as the best feature, and the selected feature from the congruence coefficient is denoted as *Y*_*k*×*o*_, where *n* > *o*.

#### 4.3.2. Kumar–Hassebrook Similarity

After selecting the best feature *Y*_*k*×*o*_ using the congruence coefficient, the Kumar–Hassebrook similarity [26] is applied on it to select the most appropriate feature. In Kumar–Hassebrook similarity, the best feature is picked by comparing the candidate feature with the target value, and the expression becomes

(3) $$\begin{array}{c} M_{KHS}=\frac{\sum TV}{\sum T^{2}+\sum V^{2}-\sum TV}\text{,} \end{array}$$

where *T* denotes the candidate feature and *V* specifies the target values. After computing the Kumar–Hassebrook similarity, the top *z* features with the highest values are selected as the final best features, and the selected feature from the Kumar–Hassebrook similarity is denoted as *V*_*k*×*z*_, where *o* > *z*.

### 4.4. Heart Disease Prediction Using SqueezeNet

The dysfunction of the actual processing of the heart is called heart disease. Generally, heart diseases are identified through various deep-learning techniques. In this research, the heart disease prediction is done using SqueezeNet [22], which is trained by the DMOA method. Here, the SqueezeNet model selects the input as *V*_*k*×*z*_ for heart disease prediction. The gain of the SqueezeNet model is that it provides better detection results with simple construction costs. Then, the structure of SqueezeNet is explained in the succeeding section.

#### 4.4.1. Structure of SqueezeNet

**Figure 2:**
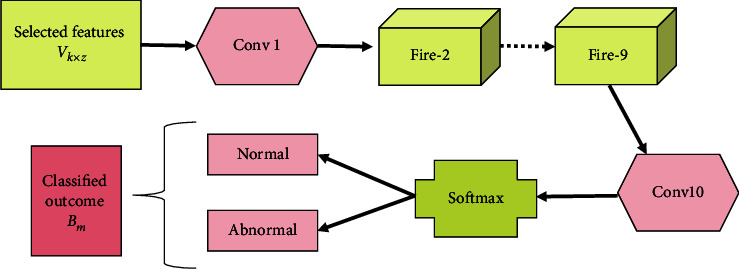
SqueezeNet design.

The SqueezeNet [22] generally comprises of various fire modules, and the fire modules contain a squeeze convolution layer and an expand layer. In the fire modules, the outcome of the squeeze convolution layer is sent to the next expand layer. Moreover, the SqueezeNet starts with a standalone convolution layer tracked by the 8 fire modules and ends with the final convolution layer. Then, the outcome of the SqueezeNet model is represented as *B*_*m*_. In addition, the SqueezeNet performs the max-pooling operation in two strides, shown in Figure 2.

#### 4.4.2. SqueezeNet Training Using DMOA

The SqueezeNet used in this research is trained with the DMOA, which is elaborated in this section. The basic principle of DMOA is based on the foraging characteristic of the dwarf mongoose. DMOA [24] is a metaheuristic model for resolving optimization complexities. DMOA has the ability to generate and improve the candidate solution for the specified optimization problems. In this algorithm, the dwarf monkeys explore the different areas of problem search space, as a result, they are moving from one food source to another. Moreover, DMO utilizes only one parameter for tuning. The algorithmic steps of DMOA are explained as follows:

*(1) Initialization*. The algorithmic constraints and solutions are initialized in the first step, which is utilized to generate the optimal solution.

*(2) Fitness Measure*. The optimal solution is chosen based on the MSE, which is formulated as

(4) $$\begin{array}{c} b_{\min}=\frac{1}{w}\overset{w}{\underset{m=1}{\sum }}{\left(B_{m}^{*}-B_{m}\right)}^{2}\text{,} \end{array}$$

where *w* denotes the total sample count, *B*_*m*_^*∗*^ denotes the expected outcome, and denotes the classified outcome of SqueezeNet.

*(3) Alpha Group*. After the population initialization, the effectiveness of the entire solution is determined. In this step, the alpha female is selected with respect to the likelihood values, which are calculated by

(5) $$\begin{array}{c} k=\frac{{\left(b_{\min}\right)}_{p}}{\sum_{p=1}^{t}{\left(b_{\min}\right)}_{p}}\text{.} \end{array}$$

Here, *t* specifies the mongoose count in *k* and *b*_min_ specifies the fitness function. The upgrading strategy of the solution is given as

(6) $$\begin{array}{c} s_{p+1}=s_{p}+\alpha_{p}*Q\text{.} \end{array}$$

Here, the distributed random number is signified as *α*_*p*_, and the vocalization of the leading female is denoted as *Q*, which sustains the family and *s*_*p*_ specifies the solution of the present iteration. After every iteration, the sleeping mount is computed, which is given by

(7) $$\begin{array}{c} L_{r}=\frac{{\left(b_{\min}\right)}_{p+1}-{\left(b_{\min}\right)}_{p}}{\max\;\left[\left|{\left(b_{\min}\right)}_{p+1}-{\left(b_{\min}\right)}_{p}\right|\right]}\text{.} \end{array}$$

Moreover, the average count of the sleeping mound is formulated as

(8) $$\begin{array}{c} \omega =\frac{\sum_{p=1}^{t}L_{r}}{t}\text{.} \end{array}$$

Here, *L*_*r*_ denotes the sleeping mount and *t* specifies the total number of sleeping mounts. After fulfilling the babysitting exchange criterion, the DMOA algorithm enters into the scouting stage.

*(4) Scout Group*. In this step, the mongoose moves in the optimal sleeping mound while the family explores in the long distance. Thus, the scout mongoose is formulated as

(9) $$\begin{array}{c} s_{p+1}=\left\{\begin{array}{cc} s_{p}-D*\alpha_{p}*z\left|s_{p}-\overset{⟶}{S}\right|\text{,} & \text{if}\omega_{p+1}>\omega_{p},\\ s_{p}+D*\alpha_{p}*z\left|s_{p}-\overset{⟶}{S}\right|\text{,} & \text{Otherwise}. \end{array}\right. \end{array}$$

Here, *z* indicates the random value among (0, 1) and then the value of *D* and $\overset{⟶}{S}$ is computed as

(10) $$\begin{array}{c} D={\left(1-\frac{U}{H_{U}}\right)}^{\left(2*U/H_{U}\right)}\text{,}\\ \overset{⟶}{S}=\overset{t}{\underset{p=1}{\sum }}\frac{s_{p*L_{r}}}{s_{p}}\text{.} \end{array}$$

Babysitters are inferior group persons so they are normally youngsters and are focused on activating the female alpha for performing the daily hunting. Algorithm 1 shows the pseudocode of DMOA.

*(5) Re-Evaluation of Feasibility*. The feasibility of the solution is determined with respect to the fitness value computation. Here, the smallest value of MSE is considered the best solution so that the poor solution is iteratively replaced by the best solution.

*(6) Termination*. All the above-mentioned processes are performed continuously till the optimum solution is attained. Algorithm 1 displays the pseudocode of the DMOA algorithm.

## 5. Results and Discussion

The results and discussion of the proposed DMOA-SqueezeNet for heart disease prediction are elucidated in this section.

### 5.1. Experimental Setup

The introduced model is implemented in the python tool on PC with windows 10 OS and intel i3 core processor.

### 5.2. Description of Dataset

The dataset used for the projected scheme is the heart disease dataset (Cleveland) [24], and the Z-Alizadeh Sani dataset [27]. The Cleveland dataset contains 76 attributes. The Z-Alizadeh Sani dataset contains a total of 303 patients record with 54 attributes. Specifically, this dataset is utilized to detect heart disease, wherein the integer values vary between 0 and 4.

### 5.3. Performance Metrics

The metrics used to assess the efficiency of DMOA-SqueezeNet are accuracy, sensitivity, and specificity, which are given in the next section.

#### 5.3.1. Accuracy

Testing accuracy is used to quantify the effectiveness of detection results, which is given by

(11) $$\begin{array}{c} u_{1}=\frac{g_{p}+g_{n}}{g_{p}+g_{n}+h_{p}+h_{n}}\text{,} \end{array}$$

where *g*_*p*_ defines the true positive, *g*_*n*_ indicates the true negative, *h*_*p*_ expresses the false positive, and *h*_*n*_ states the true negative.

#### 5.3.2. Sensitivity

The metrics used to measure the accurateness of true positive rate, which is defined by

(12) $$\begin{array}{c} u_{2}=\frac{g_{p}}{g_{p}+h_{n}}\text{.} \end{array}$$

#### 5.3.3. Specificity

The metrics used to quantify the accurateness of false negative rate, which is defined by

(13) $$\begin{array}{c} u_{3}=\frac{g_{n}}{g_{n}+h_{p}}\text{.} \end{array}$$

### 5.4. Comparative Methods

The performance of DMOA-SqueezeNet is assessed with four comparative methods, such as BF-PSO [20], bi-LSTM-CRF [4], XGBoost [21], RLNNC [1], and DMOA-SqueezeNet (without feature selection).

### 5.5. Comparative Analysis

The analysis of novel heart disease prediction is accomplished by adjusting the two types of varying data, like training data and k value.

#### 5.5.1. Analysis Regards to Cleveland Dataset

**Figure 3:**
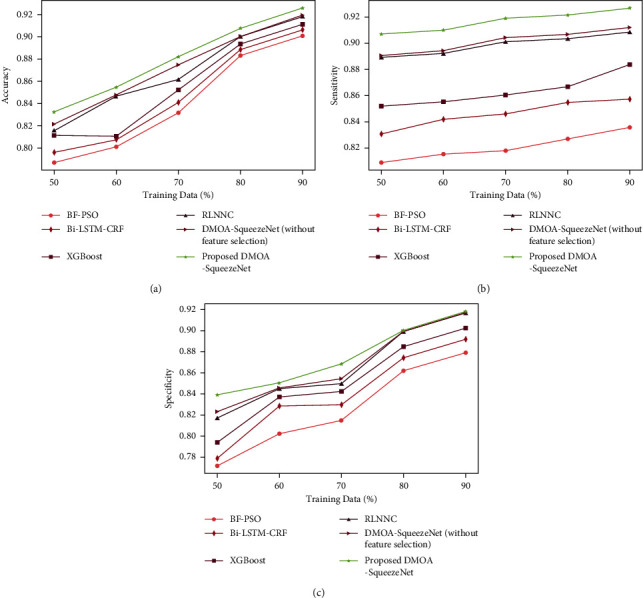
Training data-based analysis for Cleveland dataset: (a) accuracy, (b) sensitivity, and (c) specificity.

*(1) Analysis Regards to Training Data*. The comparative analysis of DMOA-SqueezeNet with varying training percentage data for the Cleveland dataset is specified in Figure 3.  Figure 3(a)) displays the accuracy graph of DMOA-SqueezeNet. The accuracy of DMOA-SqueezeNet is 0.925, which is 2.69% better than BF-PSO, 2.11% better than Bi-LSTM-CRF, 1.57% better than XGBoost, 0.831% better than RLNNC, and 0.692% better than DMOA-SqueezeNet (without feature selection) when the train data% is 90. The sensitivity graph of DMOA-SqueezeNet is exhibited in Figure 3(b)). Here, the sensitivity of DMOA-SqueezeNet is 0.926 for 90% of train data, which is 9.83%, 7.50%, 4.65%, 1.96%, and 1.6% higher than the BF-PSO, bi-LSTM-CRF, XGBoost, RLNNC, and DMOA-SqueezeNet (without feature selection), respectively. The specificity attained by the DMOA-SqueezeNet is given in Figure 3(c)). Here, the specificity of DMOA-SqueezeNet is 0.918 for 90% of train data, which is 4.29%, 2.88%, 1.72%, 0.12%, and 0.1% higher than the prevailing methods.

**Figure 4:**
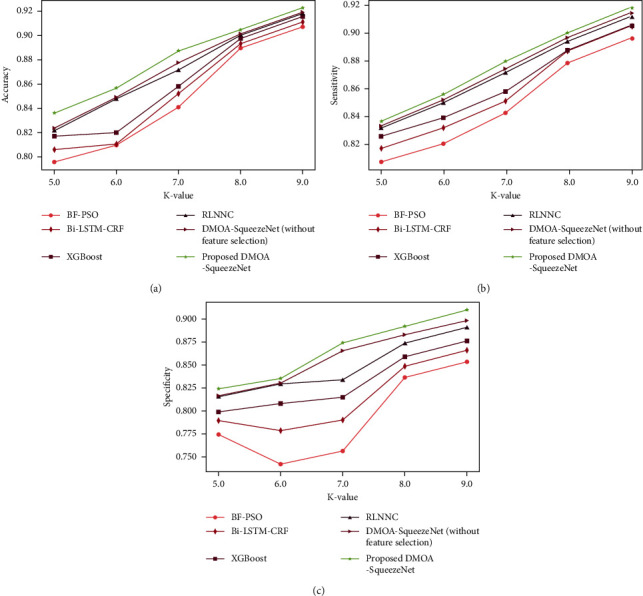
K-fold-based analysis for Cleveland dataset: (a) accuracy, (b) sensitivity, and (c) specificity.

*(2) Analysis Regards to k-value*. The accuracy graph of DMOA-SqueezeNet is exhibited in Figure 4(a)). Here, the accuracy of DMOA-SqueezeNet is 0.922 for K-Fold = 9, which is 1.66%, 1.26%, 0.80%, 0.51%, and 0.406% higher than the BF-PSO, Bi-LSTM-CRF, XGBoost, RLNNC, and DMOA-SqueezeNet (without feature selection). The sensitivity attained by the DMOA-SqueezeNet is given in Figure 4(b)). Here, the sensitivity of DMOA-SqueezeNet is 0.918 for K-Fold = 9, which is 2.41%, 1.43%, 1.40%, 0.67%, and 0.382% higher than the prevailing methods.  Figure 4(c)) shows the specificity graph of DMOA-SqueezeNet. The specificity of DMOA-SqueezeNet is 0.90, which is 6.22% better than BF-PSO, 4.83% better than bi-LSTM-CRF, 3.69% better than XGBoost, 2.08% better than RLNNC, and 1.3% better than DMOA-SqueezeNet (without feature selection) for K-Fold = 9.

#### 5.5.2. Analysis Regards to Z-Alizadeh Sani Dataset

**Figure 5:**
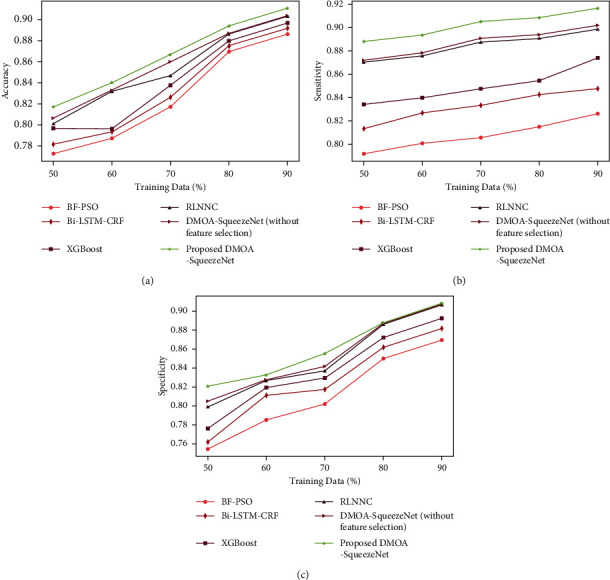
Training data-based analysis for Z-Alizadeh Sani dataset: (a) accuracy, (b) sensitivity, and (c) specificity.

*(1) Analysis Regards to Training Data*.  Figure 5 shows the comparative analysis of DMOA-SqueezeNet with varying training percentage data for the Z-Alizadeh Sani dataset.  Figure 5(a)) displays the accuracy graph of DMOA-SqueezeNet. The accuracy of DMOA-SqueezeNet is 0.911, whereas the existing BF-PSO, bi-LSTM-CRF, XGBoost, RLNNC, and DMOA-SqueezeNet (without feature selection) have an accuracy of 0.886, 0.892, 0.897, 0.903, and 0.904 when the train data% is 90. The sensitivity graph of DMOA-SqueezeNet is exhibited in Figure 5(b)). Here, the sensitivity of DMOA-SqueezeNet is 0.917 for 90% of train data, and 0.827, 0.848, 0.874, 0.899, and 0.902 for BF-PSO, bi-LSTM-CRF, XGBoost, RLNNC, and DMOA-SqueezeNet (without feature selection), respectively. The specificity attained by the DMOA-SqueezeNet is given in Figure 5(c)). Here, the specificity of DMOA-SqueezeNet is 0.908 for 90% of train data, and 0.870, 0.882, 0.893, 0.907, and 0.907 for BF-PSO, bi-LSTM-CRF, XGBoost, RLNNC, and DMOA-SqueezeNet (without feature selection), respectively.

**Figure 6:**
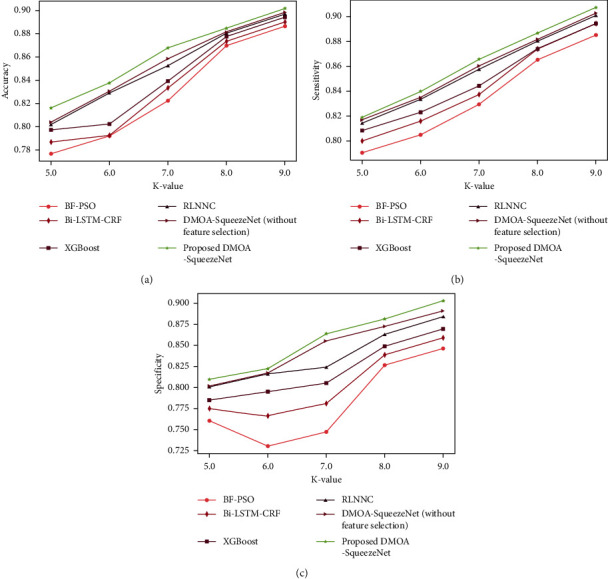
K-fold-based analysis for Z-Alizadeh Sani dataset: (a) accuracy, (b) sensitivity, (c) specificity.

*(2) Analysis Regards to k-fold*. The K-Fold analysis using the Z-Alizadeh Sani dataset is shown in Figure 6. The accuracy graph of DMOA-SqueezeNet is exhibited in Figure 6(a)). Here, the accuracy of DMOA-SqueezeNet is 0.902 for K-Fold = 9, and the existing BF-PSO, bi-LSTM-CRF, XGBoost, RLNNC, and DMOA-SqueezeNet (without feature selection) have the accuracy of 0.887, 0.890, 0.894, 0.897, and 0.898, respectively. The sensitivity attained by the DMOA-SqueezeNet is given in Figure 6(b)). Here, the sensitivity of DMOA-SqueezeNet is 0.907 for K-Fold = 9, and 0.885, 0.894, 0.894, 0.901, and 0.903 for BF-PSO, bi-LSTM-CRF, XGBoost, RLNNC, and DMOA-SqueezeNet (without feature selection).  Figure 6(c)) shows the specificity graph of DMOA-SqueezeNet. The specificity of DMOA-SqueezeNet is 0.903, and 0.846, 0.859, 0.869, 0.884, and 0.891 for BF-PSO, bi-LSTM-CRF, XGBoost, RLNNC, and DMOA-SqueezeNet (without feature selection) when K-Fold = 9.

### 5.6. Comparative Discussion

The comparative discussion of DMOA-SqueezeNet with prevailing techniques for heart disease prediction is defined in Table 1. Here, the analysis is done by varying the training data and k-value using the Cleveland dataset and Z-Alizadeh Sani dataset. In this research, for the Cleveland dataset, the DMOA-SqueezeNet acquired a superior performance than the prevailing methods based on the training data. The accuracy, sensitivity, and specificity of DMOA-SqueezeNet are 0.925, 0.926, and 0.918, whereas the prevailing methods, such as BF-PSO, are 0.900, 0.835, and 0.879, bi-LSTM-CRF is 0.906, 0.857 and 0.891, XGBoost is 0.911, 0.883 and 0.902 and RLNNC is 0.918, 0.908 and 0.917. By considering the Z-Alizadeh Sani dataset the accuracy, sensitivity, and specificity obtained by the proposed method are 0.911, 0.917, and 0.908, respectively, for varying the training data.

The reasons for the better performance of the proposed method are discussed as follows: In the proposed method, the redundant data is removed in the preprocessing step, which reduces the running time of the process. Also, the prediction process required meaningful features, which is done by the hybrid congruence coefficient Kumar–Hassebrook similarity. Moreover, the SqueezeNet model used for the prediction provides a better detection result with a simple construction cost. Thus, the performance of the proposed method is better than the conventional approaches.

## 6. Conclusion and Future Directions

The heart disease prediction technique, namely, DMOA-SqueezeNet is explicated in this research. For heart disease prediction, the input data is preprocessed, and the various methods select the appropriate features. Here, the heart disease prediction is done by the SqueezeNet model, wherein the DMOA trains the weight and bias of SqueezeNet. DMOA is modeled by adapting the feeding behavior of dwarf mongooses. Moreover, DMOA contains only one parameter for finding the optimal solution. Moreover, the preprocessing method uses quantile normalization and missing data imputation. The feature selection is done by the hybrid congruence coefficient Kumar–Hassebrook similarity. Here, the selected feature from the congruence coefficient is passed to the Kumar–Hassebrook similarity, again selecting the higher score features for heart disease prediction. Moreover, the experimental result reveals that the DMOA-SqueezeNet method attained a higher accuracy of 0.925, a sensitivity of 0.926, and a specificity of 0.918. However, the performance of the proposed method is evaluated by using some limited metrics. In the future, the effectiveness of the invented model can be progressed by adapting various optimization techniques for designing an efficient hybrid optimization scheme. Also, it will be further enhanced to classify heart diseases and the performance will be evaluated by considering more metrics.

## Figures and Tables

**Algorithm 1:**
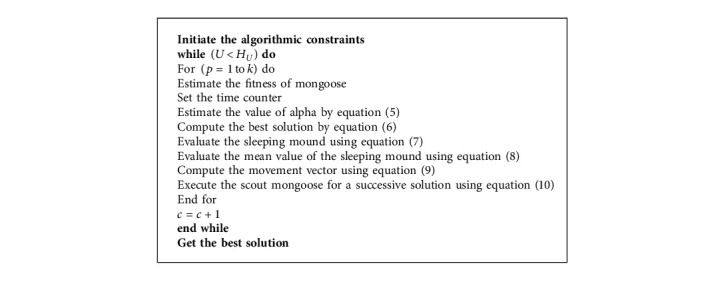
Pseudocode of DMOA.

**Table 1:** Comparative discussion.

Variations	Metrics	BF-PSO	Bi-LSTM-CRF	XGBoost	RLNNC	DMOA-SqueezeNet(without feature selection)	Proposed DMOA-SqueezeNet
*Cleveland dataset*							
Training data	Accuracy	0.900	0.906	0.911	0.918	0.919	**0.925**
	Sensitivity	0.835	0.857	0.883	0.908	0.912	**0.926**
	Specificity	0.879	0.891	0.902	0.917	0.915	**0.918**
K value	Accuracy	0.907	0.911	0.915	0.918	0.919	0.922
	Sensitivity	0.896	0.905	0.905	0.912	0.915	0.918
	Specificity	0.853	0.865	0.876	0.890	0.898	0.909

*Z-Alizadeh Sani dataset*							
Training data	Accuracy	0.886	0.892	0.897	0.903	0.904	0.911
	Sensitivity	0.827	0.848	0.874	0.899	0.902	0.917
	Specificity	0.870	0.882	0.893	0.907	0.907	0.908
K value	Accuracy	0.887	0.890	0.894	0.897	0.898	0.902
	Sensitivity	0.885	0.894	0.894	0.901	0.903	0.907
	Specificity	0.846	0.859	0.869	0.884	0.891	0.903

## Data Availability

The data used to support the findings of this study are available from the corresponding author upon request.
